# Transformational Leadership, Task-Involving Climate, and Their Implications in Male Junior Soccer Players: A Multilevel Approach

**DOI:** 10.3390/ijerph16193649

**Published:** 2019-09-28

**Authors:** Octavio Álvarez, Isabel Castillo, Vladimir Molina-García, Inés Tomás

**Affiliations:** 1 Department of Social Psychology, University of Valencia, 46010 Valencia, Spain; Octavio.Alvarez@uv.es; 2 Postgraduate and Continuing Education Centre, University of Almeria, 04120 Almeria, Spain; vmolgar@ual.es; 3 Department of Methodology of the Behavioral Sciences, University of Valencia, 46010 Valencia, Spain; Ines.Tomas@uv.es

**Keywords:** transformational leadership, motivational climate, soccer, junior players, multilevel SEM

## Abstract

Despite the well-known positive consequences of transformational coaches in sport, there is still little research exploring the mechanisms through which coaches’ transformational leadership exerts its impact on athletes. Multilevel SEM was used to examine the relationship between coaches’ transformational leadership style, a task-involving climate, and leadership effectiveness outcome criteria (i.e., players’ extra effort, coach effectiveness, and satisfaction with their coach), separately estimating between and within effects. A representative sample of 625 Spanish male soccer players ranging from 16 to 18 years old and nested in 50 teams completed a questionnaire package tapping the variables of interest. Results confirmed that at the team level, team perceptions of transformational leadership positively predicted teams’ perceptions of task climate, which in turn positively predicted the three outcome criteria. At the individual level, players’ perceptions of transformational leadership positively predicted teams’ perceptions of task climate, which in turn positively predicted teams’ extra effort and coach effectiveness. Mediation effects appeared at the team level for all the outcome criteria, and at the individual only for extra effort. Transformational leadership is recommended to enhance task climate, in order to increase players’ extra effort, their perceptions of the effectiveness of their coach, and their satisfaction with his/her leadership style.

## 1. Introduction

The transformational leadership paradigm [1] has been widely used to understand the effects of leaders’ behaviors in the field of applied psychology [2], and its significant potential for research in the sporting context has also been increasingly recognized [3,4,5].

Transformational leaders (i.e., coaches) became a behavioral model for followers (i.e., athletes) (idealize influence) to stimulate them to think in different ways to face new and old challenges and issues (intellectual stimulation), give them challenges and meaning in everyday activities (inspiration motivation), and recognize individual differences through a supportive leadership style (individualized consideration). Through transformational leadership behaviors, coaches get results by going further than even the team members expect, improving team outcomes through their leadership (e.g., [3,6]), and fostering not only better coaches but also better athletes [4].

The coach plays a key role in the sports team. He/she articulates the power balance in the team, leads the team toward the targets, coordinates and creates cohesion among team members, and acts as a cornerstone in the construction of motivational climates and team performance. Moreover, the coach has the role of communicating and establishing relations with every team member, and he/she is the link between the team and the other elements (managers, media, etc.) [3].

There is a consensus that there is more than one effective way to lead a team and that leaders can attend to both their team and individual members simultaneously [7]. Specifically, the transformational leadership construct comprises behaviors targeted at both the team (i.e., idealized influence and inspirational motivation) and individual (i.e., intellectual stimulation and individual consideration) levels. Nevertheless, research has shown that the two leadership processes (attending to each team member as an individual and attending to a team as a whole) are independent from each other (e.g., [8]) and, consequently, may have different outcomes (e.g., [9]).

Transformational leadership in sport has been shown to be positively related to several outcomes, including self-competence, enjoyment, and collective efficacy [10], self-efficacy [11], positive experiences related to physical activity and sport [12,13], extra effort [14], self-determined motivation [11,12,15], sport performance [16,17], and coaching competency and athlete satisfaction [18], among others (see [3] for a review). The most studied criteria in transformational leadership have been extra effort, leader effectiveness, and leadership satisfaction [6,19,20,21]. Although the literature provides information on athlete outcomes that may be associated with coaches’ use of transformational leadership, few studies have evaluated the processes through which transformational leadership exerts its influence on athletes’ outcomes [5]. Accordingly, several studies (e.g., [11,17]) have indicated that transformational leaders may positively influence follower outcomes by creating environments focused on learning from the task, that is, through motivation-based mechanisms. As such, it is strongly encouraged to explore whether transformational leaders are more likely to foster a task-involving motivational climate and how this climate might influence athletes.

### 1.1. Motivational Climate

The motivational climate has been identified as a construct that can significantly influence athletes’ behaviors and achievement strategies. Based on the achievement goal theory (AGT; [22,23]), motivational climate emphasizes the central role of the social context in motivational processes, and it is defined as the individual’s perceptions of the goal situational structure in an achievement context. Ames [22] distinguished between contexts that emphasize self-referenced criteria for success (task-involving climate) and those that reinforce social comparison through the promotion of norm-referenced criteria for success (ego-involving climate).

In the sport context, motivational climate refers to the individual athletes’ perceptions of the way their coaches define success or failure and how they value competence in practice and games [22,24]. An ego-involving climate is characterized by athletes’ perceptions that they are systematically punished for their mistakes. The most skilled athletes are the most recognized, and teammates try to outperform each other. By contrast, a task-involving climate is characterized by athletes’ perceptions that mistakes are part of the improvement process. Self-reference, instead of comparison with others, is the way to measure improvement, and every team member is recognized for his/her effort and task involvement [25].

According to Duda and Balaguer [26], research on motivational climate in the sport context suggests that creating a task climate has positive effects on athletes, whereas creating an ego climate results in negative sports experiences. For example, task climate has been positively related to greater enjoyment, satisfaction, positive affect, wellbeing, effort, intrinsic motivation, and satisfaction of basic needs (autonomy, competence, and relatedness) (e.g., [26,27,28,29]). By contrast, an ego climate has been positively related to negative outcomes such as anxiety, low focus, poor performance, and burnout (e.g., [30,31]). In this research, we focus on task climate as a positive climate environment.

### 1.2. Leadership and Motivational Climates

Duda and Balaguer’s [32] integrated model of the coach’s influence on motivational processes suggested that there is a strong relationship between the athletes’ perceptions of the coach’s leadership style and motivational factors. They offered a framework that integrates aspects of the mediational model of leadership [33] and the multidimensional model of leadership [34,35] with variables from the AGT [22,23]. To this end, Duda and Balaguer integrated dispositional and climate motivational factors as preferred and real coach behaviors. They concluded that coaches who are more involved in instruction and the athletes’ wellbeing achieve teams with higher levels of task motivational climate. Hence, leadership behaviors can be considered determinants of motivational climate. Interestingly, Álvarez and colleagues [16], in an intervention with a senior rhythmic gymnastics team, suggested that increases in the coach’s transformational leadership style and the athletes’ perceptions of task climate were related to improvements in team performance. Recently, Kao and Watson II [36], in a sample of basketball players, reported that coaches’ transformational leadership was positively related to perceived mastery climate at the individual and group levels. Additionally, Newland and colleagues [37] suggested the importance of including explorations of motivational climates as mediators in the relationships between transformational leadership and positive player outcomes.

**Figure 1:**
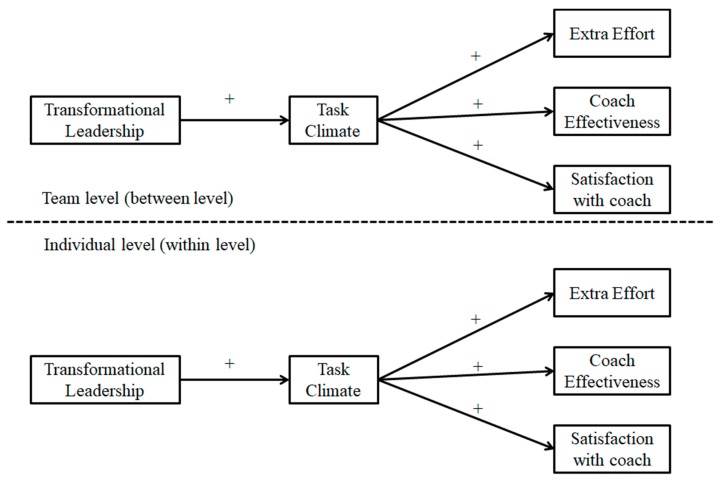
Hypothesized multilevel SEM model (M1) of the associations between transformational leadership style, task-involving climate, and outcome criteria.

In sum, task motivational climate enhances athletes’ wellbeing, performance, and positive experiences in sports practice, and it promotes good results in athletes’ performance. Moreover, these good results improve when the coach employs transformational leadership [3]. Therefore, based on the suggestion that there is a strong relationship between coaches’ leadership styles and motivational factors [32], the fact that players are nested within teams, and previous research [16,36], the purpose of the present study was to examine the relationship between coaches’ transformational leadership, a task-involving climate, and effective leadership outcomes (extra effort, coach effectiveness, and satisfaction with the coach), testing the mediational role of a task-involving motivational climate in the relationship between coaches’ leadership style and outcome criteria, at the individual level (within-level effects) and, more importantly, at the soccer team level (between-level effects). The following hypotheses were formulated: (1) transformational leadership will positively predict a task-involving climate; (2) a task-involving climate will positively predict the outcome variables; and (3) a task-involving climate will mediate the relationship between players’ perceptions of their coaches’ leadership style and the outcome variables (see Figure 1).

## 2. Materials and Methods

### 2.1. Participants

Participants were recruited from 50 junior federated soccer teams from Granada Province, Spain. All the soccer teams were coached by males (N = 50; M age = 38.76 ± 9.39; M experience = 7.52 years of coaching, SD experience = 1.12). The majority (74.9%) had been federated for five years or more, and only 3.2% were in their first year as federated soccer players. In all, 625 male soccer players from 16 to 18 years old (M = 16.87; SD = 0.81) were approached to complete a representative sample of the universe of reference, which represented 47.21% and a sample error of 0.03.

### 2.2. Measures

Players’ perceptions of coaches’ transformational leadership style were assessed using the transformational leadership subscale of the Spanish version, adapted to the sport context [6], of the Multifactorial Leadership Questionnaire MLQ-5X^©^, [38]. A total of 20 items were selected that assessed transformational leadership in four dimensions: idealized influence (8 items), inspirational motivation (4 items), intellectual stimulation (4 items), and individualized consideration (4 items). The MLQ-5X questionnaire started with the stem: “My coach…” and participants responded on a 5-point Likert scale (1 = never; 5 = always). An example item is (my coach) “treats me as an individual rather than just a member of the team”. Previous studies found that the items in the transformational dimensions tend to converge in a single dimension (e.g., [37]). For this research, we used a general indicator of the transformational leadership style consisting of the mean of all the transformational behaviors. This instrument is under copyright and available from Mind Garden (www.mindgarden.com). Previous sports research (e.g., in taekwondo) has supported the internal reliability of this scale (e.g., [6]), with Cronbach’s alpha above 0.89.

Player perception of a task-involving climate was assessed with the task climate subscale of the Spanish version of the Perceived Motivational Climate in Sport Questionnaire-2 PMSCQ-2; [25], adapted to soccer [39]. This subscale is composed of 15 items examining the degree to which the climate created by the coach was deemed to be more or less a task climate (e.g., “each player contributes in some important way”). All the sentences are preceded by the stem “On my soccer team…”, and responses are provided on a 5-point Likert-type scale ranging from 1 (strongly disagree) to 5 (strongly agree). Previous sport studies at different competitive levels and in different sports have confirmed the adequate internal reliability of this subscale (e.g., [25,39]), with Cronbach’s alpha ranging from 0.80 to 0.88.

The outcome variables were measured using nine items from the MLQ-5X questionnaire, described above, corresponding to the following three subscales: extra effort (3 items, e.g., “gets me to do more than I expected to do”), coach effectiveness (4 items, e.g., “is effective in meeting my sport-related needs”), and satisfaction with their coach (2 items, e.g., “works with me in a satisfactory way”). Previous sport studies (e.g., involving taekwondo players) have shown adequate internal reliability for these subscales (e.g., [6]), with Cronbach’s alpha ranging from 0.74 to 0.78.

### 2.3. Procedure

The director of each soccer club was contacted via e-mail. The message contained the aim of the study and requested that their players volunteer as participants. Once the clubs’ directors had expressed interest in participating in the study, a researcher visited the clubs, distributed the questionnaires, and provided instructions in a classroom setting selected by the club. After providing clubs and players informed consent, players completed the questionnaire anonymously and voluntarily in about 30 min, together with their respective teams. Data were collected at the beginning or end of a training session during the first part of the sports season (between September and November) to allow time for the coach implemented transformational behaviors and created task motivational climate to be established. The present research was reviewed and approved by the ethics committee of the university of the first author (Ref: H1465763942085).

### 2.4. Statistical Analyses

Statistical analyses were performed with IBM SPSS Statistics version 20 (IBM Corp., Armonk, NY, USA) and Mplus version 8.1 [40] using robust maximum likelihood estimation. For all variables, less than 5% of values were missing. Confirmatory factor analyses (CFA) were conducted to check the factorial structure of the questionnaires. Internal consistency of the items on the composite scale of leadership, task-involving motivational climate, and outcome variables was tested with Cronbach’s alpha, composite reliability (rho), and the average variance extracted (AVE). Alpha and rho values of 0.70 or greater indicate an acceptable reliability, and AVE values of 0.50 or greater indicate a good score reliability. Means, standard deviations, and correlations were used to describe the studied variables. Multilevel structural equation modeling (MSEM) was employed to assess the hypothesized structural model. Several fit indices, including chi-square, comparative fit index (CFI), Tucker–Lewis index (TLI), root mean square error of approximation (RMSEA), and standardized root mean residuals (SRMR), were employed to consider the adequacy of the CFA models and the hypothesized MSEM model. Values of CFI and TLI > 0.90 and values of RMSEA and SRMR ≤ 0.10 were applied to indicate adequate fit.

Prior to running the MSEM analyses, average deviation indexes ADI; [41] were computed and analyzed for each of the three scales to ensure within-team agreement. Because all of the scales used a 5-point Likert response scale, the cut-off value for the ADI was 0.83 [42]; therefore, we concluded that there was within-team agreement when the ADI values were ≤ 0.83. Additionally, we tested between-team discrimination of the study variables by using one-way analysis of variance (ANOVA).

Finally, we used Monte Carlo (MC) confidence intervals (CI) to test the significance of the indirect effects by using the web utility provided by Selig and Preacher [43]. If the confidence interval does not include zero, the null hypothesis of no mediation is rejected, confirming the mediation effect.

## 3. Results

### 3.1. CFA, Descriptive Statistics, and Reliability

The CFA results revealed that the proposed factorial structure was acceptable for each instrument of the study: a one-factor model solution for transformational leadership style (χ2 (164) = 532.22, *p* < 0.01; RMSEA = 0.060 (CI = 0.054–0.065); SRMR = 0.046; TLI = 0.959; CFI = 0.964) and for task-involving climate (χ2 (77) = 499.39, *p* < 0.01; RMSEA = 0.074 (CI = 0.070–0.079); SRMR = 0.058; TLI = 0.931; CFI = 0.942), and a three-factor model solution for outcome criteria (χ2 (24) = 99.85, *p* < 0.01; RMSEA = 0.071 (CI = 0.057–0.085); SRMR = 0.034; TLI = 0.971; CFI = 0.981). All scales were found to have an acceptable model fit, and the standardized factor loadings for all the items in their designated factors were greater than 0.40 (*p* < 0.001) and indicated that no items should be dropped. For reasons of brevity, the results of the CFAs are not presented here, but they are available from the first author on request.

Descriptive statistics (mean and standard deviation), internal reliability scores, and the correlation matrix for the study variables are presented in Table 1. All the study variables were significantly and positively correlated. The internal reliability coefficients for all the scales were adequate. If AVE is less than 0.50, but rho is higher than 0.60, the convergent validity of the construct can be considered still adequate [44].

### 3.2. Multilevel Analyses

The results showed that the ADI value was below the proposed cut off of 0.83 for the transformational leadership scale (M = 0.69, SD = 0.09), the task-involving climate scale (M = 0.67, SD = 0.12), the extra effort scale (M = 0.70, SD = 0.15), the coach effectiveness scale (M = 0.67, SD = 0.13), and the satisfaction with the coach scale (M = 0.69, SD = 0.16), indicating that there was within-team agreement in the study variables. The results of the one-way ANOVA carried out for each of the variables included in the model indicated significant differences in the team’s scores for transformational leadership style (F(49,575) = 4.07, *p* < 0.01), task climate (F(49,575) = 2.45, *p* < 0.01), extra effort (F(49,575) = 2.71, *p* < 0.01), coach effectiveness (F(49,575) = 3.03, *p* < 0.01), and satisfaction with the coach (F(49,568) = 2.18, *p* < 0.01). These results provided reasonable justification for further multilevel analyses, that is, testing the hypothetical mediation model (see Figure 1) at both levels (individual and team level).

**Figure 2:**
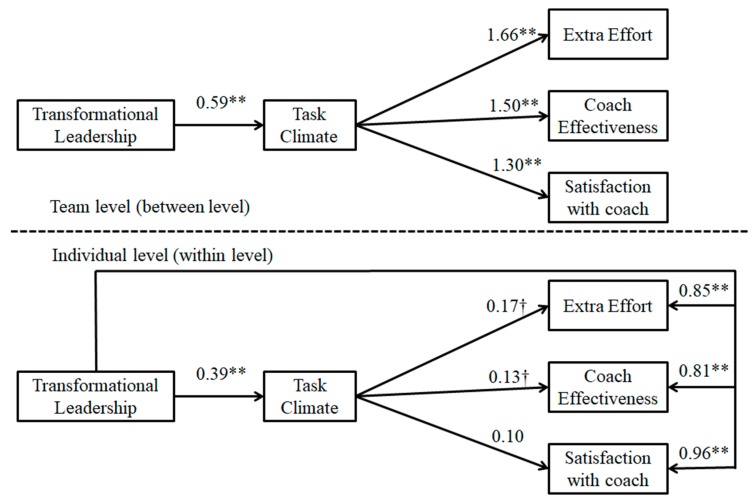
Unstandardized solution for the revised (M2) mediation multilevel SEM of the associations between transformational leadership style, task-involving climate, and outcome criteria. † *p* < 0.10; ** *p* < 0.01.

The MSEM indicated that the hypothesized model (M1) did not show adequate fit to the data (χ2 (9) = 371.12, *p* < 0.01; RMSEA = 0.254; CFI = 0.791; TLI = 0.376; SRMR-within = 0.223; SRMR-between = 0.191). Modification indexes suggested adding a parameter between transformation leadership and outcome criteria at the individual level. The MSEM for the revised model (M2) showed adequate fit to the data: χ2 (6) = 16.62, *p* < 0.01; RMSEA = 0.053; CFI = 0.992; TLI = 0.973; SRMR-within = 0.013; SRMR-between = 0.141. Regarding within-effects (see Figure 2), results revealed that players’ perceptions of transformational leadership style were positively associated with task climate (B = 0.39, *p* = 0.001) and the three outcome criteria (i.e., extra effort (B = 0.85, *p* = 0.001), coach effectiveness (B = 0.81, *p* = 0.001), and satisfaction with the coach (B = 0.96, *p* = 0.001)). In turn, task climate was positively and marginally associated with extra effort (B = 0.17, *p* = 0.06) and coach effectiveness (B = 0.13, *p* = 0.08); and in contrast to what was expected, task climate did not show a significant association with satisfaction with the coach (B = 0.10, *p* = 0.31). With regard to between-effects (see Figure 2), results revealed that players’ shared perceptions of the transformational leadership style were positively associated with task climate (B = 0.59, *p* = 0.001), which in turn was positively associated with the three outcome criteria (i.e., extra effort (B = 1.66, *p* = 0.001), coach effectiveness (B = 1.50, *p* = 0.001), and satisfaction with the coach (B = 1.30, *p* = 0.001)).

### 3.3. Mediation Effects

Taking into account the MSEM results, three between indirect effects (IE) were identified at the team level. Results suggested that task climate mediated the relationship between the transformational leadership style and the three outcome criteria at the team level: extra effort (IE = 0.98, 95% MC CI = [0.37, 1.59]), coach effectiveness (IE = 0.89, 95% MC CI = [0.37, 1.41]), and satisfaction with the coach (IE = 0.77, 95% MC CI = [0.07, 1.47]).

At the individual level, results suggested that task climate mediated the relationship between the transformational leadership style and extra effort (IE = 0.07, 95% MC CI = [0.01, 0.13]). However, no significant within mediation effects were identified for coach effectiveness (IE = 0.05, 95% MC CI = [−0.01, 0.10]) or satisfaction with the coach (IE = 0.04, 95% MC CI = [−0.04, 0.12]).

## 4. Discussion

Based on transformational theory and AGT, this study examined the associations between players’ perceptions of coaches’ transformational leadership style, a task-involving motivational climate, and outcome variables (players’ extra effort, perceived coach effectiveness, and satisfaction with the coach). In addition, we tested the mediational role of the task-involving motivational climate in the relationships between coaches’ leadership style and the outcome criteria, and we analyzed the relationships at both the individual and team levels. Papaioannou and colleagues [45] pointed out that, although the motivational climate is an inherent group-level variable, the responses can be considered at both the individual and group levels.

In accordance with our first hypothesis, study results showed a positive association between a transformational leadership style and task climate at both the individual and team levels. Players and teams that perceived a transformational leadership style from their coaches were also more likely to perceive a task-involving climate. These results extend the findings of Kao and Watson II [36], and they confirm that both individual and team perceptions of a transformational leadership style were associated with a positive and adaptive motivational climate.

Hodge and colleagues [46] recommended a transformational leadership style and task climate to obtain the best outcomes from a team. Our results indicated that, at the team level, perceptions of a task-involving climate played a direct role in the outcome criteria and a mediational role between the transformational leadership style and outcome criteria, confirming hypotheses two and three. In practical terms, teams that viewed coaches as creating a task-involving climate also perceived extra effort, higher coach effectiveness, and greater satisfaction with their coaches; additionally, team perceptions of a transformational leadership style were associated with the outcome criteria through a task-involving climate. Whereas a task-involving climate has an influence on outcome criteria at the team level, at the individual level, the task climate influence was marginal and only affected players’ extra effort and leader effectiveness. Results also suggested that, at the individual level, task climate mediated the relationship between the transformational leadership style and extra effort.

Results of this research reinforce the proposal of Wu and colleagues [8], who suggested that the mechanisms of influence of the transformational leadership style are different on groups as a whole and on the individuals within groups. The results also corroborate that the establishment of a positive motivational climate at the team level is not perceived as strong at the individual level [12]. These results suggest that the coach’s dyadic relationship with each member of the team (individual level) is enough for the athlete to make extra effort and perceive more satisfaction with and effectiveness of his/her coach, whereas, at the group level, transformational leadership’s influence is fully mediated by the task-involving climate. In other words, the positive influence of the transformational leadership style on team effort, team coach satisfaction, and team coach effectiveness is indirect through the perceptions of a task-involving coaching climate, confirming the group nature of motivational climates [45,47] and distinguishing leadership from the motivational atmosphere where the influence takes place.

The positive relationship between the task-involving motivational climate and the outcome criteria tested in our study was supported by previous studies that confirmed positive experiences related to task climates (e.g., [39,48,49]). Specifically, our results are aligned with Duda and Balaguer [32], who suggested a positive relationship between task climate involvement and desired outcomes for participants. Additionally, Vella and colleagues [13,50] recommended the coach’s transformational style to enhance positive experiences in sport.

## 5. Conclusions, Limitations and Practical Implications

This study confirms that the task involvement climate is a positive and appropriate motivational climate, as proposed in the literature reviewed [39,48,49]. Additionally, the transformational leadership style promoted good motivational climates (task climate) and extra effort in athletes, athletes’ perceptions of coach effectiveness, and satisfaction with the coach’s leadership. That is, coach’s display a transformational leadership style by showing determination and confidence in their team, having fluent and frequent dialogues with players, stimulating athletes to participate and communicate their thoughts and feelings about the team and individual performance, and creating a task-involving climate. In doing so, they emphasize a self-referenced concept of ability focused on effort and learning, and their players tend to make extra effort, perceive their coach as more effective, and be more satisfied with their coach’s leadership. In sum, using a transformational leadership style effectively has better personal outcomes and improves the team as a whole by creating a task-involving motivational climate.

Some limitations of this study include the fact that the sample is made up only of young male soccer players, the information is obtained through self-reported measures, and the study design is cross-sectional. A longitudinal design is required to offer conclusions regarding the causal effects of the transformational leadership and task climate on the targeted outcomes. Future research should, therefore, include a representative sample of male and female soccer players to test for gender invariance, or to generalize findings to female samples. In future studies, it would be advisable to include the use of some observational measures or objective measures of players’ effort. These limitations lead us to interpret the results with caution. Despite the limitations, advantages of this study include its multilevel design with a representative sample of the universe of reference in Spain, grounded in an integrated model of coaching leadership and motivational climate [32] using the Transformational leadership theory [1] and Achievement goal theory [22,23].

From an applied perspective, we emphasize the need for federated coach training programs that include psychosocial issues and, more specifically, transformational leadership behaviors, as well as task-involving climates. The coach plays a crucial role in the development of positive/negative experiences in athletic sports practice, and official institutions have the responsibility of helping them by providing specific tools and skills to carry out their mission.

## Figures and Tables

**Table 1:** Descriptive statistics, internal consistency, and Pearson correlation coefficients for the study variables.

Variables	Mean	*SD*	Alpha	rho	AVE	1	2	3	4	5
1.Transformational leadership	2.52	0.54	0.87	0.91	0.52	-				
2. Task climate	3.83	0.57	0.86	0.94	0.51	0.40 **	-			
3. Extra effort	2.67	0.82	0.69	0.73	0.47	0.62 **	0.38 **	-		
4. Coach effectiveness	2.67	0.69	0.66	0.70	0.47	0.67 **	0.39 **	0.69 **	-	
5. Satisfaction with coach	2.58	0.84	0.59^+^	0.63	0.46	0.63 **	0.32 **	0.60 **	0.62 **	-

*Note*. Range = 1–5. ** *p* < 0.01; + Pearson’s correlation is reported because this construct was assessed by two items.
